# Resources for health literacy among caregivers of prematurely born children: a scoping review

**DOI:** 10.1590/0034-7167-2023-0062

**Published:** 2024-03-15

**Authors:** Ana Clara Gomes Andrade, Giovanna Barbosa Mendes, Mariana Fuentes Mendoza Rodrigues Soares, Suelen Rosa de Oliveira, Luciano Marques dos Santos, Elysângela Dittz Duarte

**Affiliations:** I Universidade Federal de Minas Gerais. Belo Horizonte, Minas Gerais, Brazil; II Universidade Estadual de Feira de Santana. Feira de Santana, Bahia, Brazil

**Keywords:** Health Literacy, Family, Caregivers, Ambulatory Care, Infant, Premature, Alfabetización en Salud, Familia, Cuidadores, Atención Ambulatoria, Recién Nacido Prematuro, Letramento em Saúde, Família, Cuidadores, Assistência Ambulatorial, Recém-Nascido Prematuro

## Abstract

**Objectives:**

to map the available evidence on resources used to promote health literacy among caregivers of prematurely born children during outpatient follow-up.

**Methods:**

the Joanna Briggs Institute’s scope review protocol was utilized. The search encompassed six databases, incorporating studies from 2012 to 2022.

**Results:**

the three included publications revealed that the resources employed are: mobile applications, phone calls, individual counseling, videos, educational pamphlets, and group discussions. Implementing an education protocol during the transition home enhances scientifically grounded health promotion rates.

**Conclusions:**

there is limited literature addressing the health literacy of these caregivers. The nursing team plays a crucial role in health education and in developing resources applicable to these families.

## RESUMO

**Objetivos:**

mapear as evidências disponíveis sobre os recursos utilizados para a promoção do letramento em saúde de cuidadores de crianças nascidas prematuras no seguimento ambulatorial.

**Métodos:**

utilizou-se o protocolo de revisão de escopo do *Joanna Briggs Institute*. A busca foi realizada em seis bases de dados, incluindo estudos entre 2012 e 2022.

**Resultados:**

as três publicações incluídas evidenciaram que os recursos utilizados são: aplicativos para celulares, ligações telefônicas, aconselhamento individual, vídeos e folhetos educativos e discussões em grupos. Efetivar um protocolo de educação na transição para casa aumenta os índices de promoção da saúde cientificamente embasada.

**Conclusões:**

pouco se aborda na literatura sobre o letramento em saúde desses cuidadores. A equipe de enfermagem desempenha um papel fundamental na educação em saúde e na construção de recursos que podem ser aplicados a essas famílias.

## RESUMEN

**Objetivos:**

mapear las evidencias disponibles sobre los recursos utilizados para la promoción del alfabetismo en salud de cuidadores de niños nacidos prematuramente en el seguimiento ambulatorio.

**Métodos:**

se utilizó el protocolo de revisión de alcance del *Joanna Briggs Institute*. La búsqueda se realizó en seis bases de datos, incluyendo estudios entre 2012 y 2022.

**Resultados:**

las tres publicaciones incluidas evidenciaron que los recursos utilizados son: aplicaciones para teléfonos móviles, llamadas telefónicas, asesoramiento individual, videos y folletos educativos, y discusiones en grupos. Implementar un protocolo educativo en la transición al hogar aumenta los índices de promoción de la salud respaldada científicamente.

**Conclusiones:**

hay poca información en la literatura sobre el alfabetismo en salud de estos cuidadores. El equipo de enfermería juega un papel fundamental en la educación en salud y en la creación de recursos que pueden aplicarse a estas familias.

## INTRODUCTION

Health literacy involves a set of cognitive and social competencies that empower individuals to access, understand, and use information to promote and maintain good health. It is an essential component of health education^(1)^. The development of these competencies can contribute to better caregiving practices by parents and other caregivers of at-risk children, including prematurely born children^(2)^.

Premature birth, defined as occurring before completing 37 weeks of gestation, is the leading cause of death for children under 5 years old, categorizing them as at-risk children^(3)^. To promote the optimal development and growth of these children, it is necessary to consider and investigate the social determinants of health, such as the socioeconomic and cultural context in which these children are embedded^(4)^.

The repercussions of prematurity on children’s health^(5)^ and the demands for continuous care from professionals and family members are well-known^(6-7)^. Therefore, in addition to specialized services, there is a significant demand for families and/or caregivers providing care at home. To perform this care effectively, family members and/or caregivers need to understand the unique needs of these children^(8)^, which differ from those commonly required by same-aged children without health conditions.

A study conducted with mothers of children under two years old in three Family Health Units (UBSF) in the municipality of Matinhas, state of Paraíba, Brazil, identified several factors emphasizing the importance of monitoring a child’s development during pediatric check-ups. Respondents reported that monitoring increased participation in consultations, autonomy, exchange of experiences with other mothers, and promoted confidence in caring for their child. They also emphasized that professional guidance establishes trust and a bond with the Family Health Team (ESF)^(9)^, facilitating the planned follow-up according to the public health policies of the Unified Health System (SUS)^(10)^.

In the Brazilian context, the acknowledgment of the need for specialized care for prematurely born children is embodied in the Technical Note for organizing the healthcare network. It focuses on primary healthcare and specialized outpatient care, defining strategies for the follow-up of high-risk newborns after hospital discharge^(4)^. According to this publication, follow-up should be carried out collaboratively between specialized care and primary care, focusing on clinical stabilization, surveillance for early detection of complications due to risk factors and identified morbidities, strengthening the family’s caregiving capacity, and providing direct support to the child and their family^(4)^. The outpatient setting provides an opportunity for early diagnosis of growth and development abnormalities, referral for specialized care when necessary, and support for parents to ensure proper care for their children^(11)^.

In the context of caregiver participation in care, it is essential that health education actions aim to achieve health literacy. This statement is based on the understanding that health literacy is a concept guiding the pedagogical approach of health education actions and is also a key indicator of achieving results when an action is implemented^(12)^. Although it has an individual perspective, health literacy can be a determining factor for population health by facilitating decision-making to meet health needs^(13)^. Therefore, it has the potential to contribute to the development of caregiving skills and knowledge of the health condition for safe home care.

A study conducted with 234 Brazilians diagnosed with Arterial Hypertension revealed that better results in adherence to pharmacological treatment were related to a higher ability to understand numerical instructions and reading^(14)^. Other studies^(15-16)^ highlight the benefits of health literacy, such as improvement in cancer treatment and decision-making in breast cancer patients, as well as the reduction of anxiety levels during the process. Furthermore, it was found that patients with low health literacy have difficulties understanding the types of treatment, asking questions, and monitoring their care^(17)^. Literacy provides the development and enhancement of skills, enabling caregivers to have more confidence in child care^(18)^.

Although there is a growing number of findings in the national and international scientific literature demonstrating the contributions of health literacy to individual care, no studies were identified that specifically aimed to improve the health literacy of family members and other caregivers of at-risk children. This does not allow us to assert the absence of actions that promote the development of literacy in the context of health education.

Considering the above, we recognize the importance of identifying, in the national and international literature, actions directed towards caregivers of at-risk children that aim to qualify the care provided and analyzing them based on the concept of health literacy. This will enable the identification of health education actions with the potential to promote health literacy.

## OBJECTIVES

To map the available evidence on resources used to promote literacy among caregivers of prematurely born children within the scope of ambulatory follow-ups.

## METHODS

### Ethical aspects

As this is a review study, approval from the Research Ethics Committee was waived.

### Study type

This is a scoping review that allows identifying the extent and nature of evidence on a specific subject^(19)^. The Joanna Briggs Institute’s (JBI) scoping review protocol^(20)^ and the Preferred Reporting Items for Systematic Reviews and Meta-Analyses Extension for Scoping Reviews (PRISMA-ScR) checklist^(21)^ were used to guide the study’s conduct and writing.

### Methodological procedures

The objectives, inclusion criteria, and methods for this scoping review were specified in advance and documented in a scoping review protocol developed according to the JBI-guided protocol^(20)^. The protocol was submitted to the Open Science Framework (OSF) platform and can be accessed at https://osf.io/da5rs/.

The scoping review questions were formulated based on the Population, Concept, and Context (PCC) mnemonic to achieve the previously described objective for this review (Chart 1). The main question for this review is: What resources are being used for health literacy (concept) of caregivers of prematurely born children (population) in ambulatory follow-ups (context)?

**Chart 1:** Data extraction categories, Belo Horizonte, Minas Gerais, Brazil, 2023

Population	Concept	Context
Sample size Health condition Socioeconomic characteristics (education, income) Demographic characteristics of caregivers and children Literacy definition	Resources used for literacy Approach to literacy Geographic origin of the study	Settings (outpatient clinics, hospitals) Data collection methods

Secondary questions were formulated for a better understanding of PCC and to support the answer to the main question. These include: What are the characteristics of the studied population? What methodological approaches were used to promote literacy among caregivers? What results were obtained with the interventions conducted? How was literacy defined by the researchers? For this study, the concept of health literacy proposed by the World Health Organization was used^(1)^.

Inclusion criteria for study selection were: studies with primary caregivers of prematurely born children; addressing actions implemented to increase caregivers’ knowledge and its use in modifying care or developing instruments for this purpose; original research with qualitative, quantitative, mixed methods, or intervention approaches; peer-reviewed journals; case studies; publications in English, Portuguese, and Spanish; studies published between 2012 and 2022. Excluded publications were those with only abstract availability; editorials and opinion articles; literature reviews; case reports; gray literature.

### Data collection and organization

The search strategy was conducted by combining the descriptors “Premature” and “Family” (Population), Health Literacy (Concept), and Ambulatory Care (Context), in Portuguese, English, or Spanish, according to each database, as shown in Chart 2.

**Chart 2:** Search strategies, Belo Horizonte, Minas Gerais, Brazil, 2023

Data Base	Search Strategies
*Medline/PubMed*	((((((“Health Literacy”[Mesh]) OR (“Health Literacy”[Title/Abstract])) OR (“Health Education”[Mesh])) OR (“Health Education”[Title/Abstract])) AND ((“Family”[Mesh]) OR (Family[Title/Abstract]))) AND (((“Ambulatory Care”[Mesh]) OR (“Ambulatory Care”[Title/Abstract])) OR (Follow up[Title/Abstract]))) AND ((“Infant, Premature”[Mesh]) OR (“Premature Infant”[Title/Abstract] OR “Premature Infants”[Title/Abstract] OR “Preterm Infant”[Title/Abstract] OR “Preterm Infants”[Title/Abstract]))
*Embase*	(‘health literacy’/exp OR ‘health literacy’ OR ‘health education’/exp OR ‘health education’) AND (‘family’/exp OR family) AND (‘ambulatory care’/exp OR ‘ambulatory care’ OR ‘follow up’/exp OR ‘follow up’) AND (‘prematurity’/exp OR prematurity)
*Cochrane*	(“Health Literacy” OR “Health Education”) AND (Family) AND (“Ambulatory Care” OR “Follow up”) AND (“Infant, Premature” OR “Premature Infant” OR “Premature Infants” OR “Preterm Infant” OR “Preterm Infants”)
*Scopus*	TITLE-ABS-KEY ((“Health Literacy” OR “Health Education”) AND (Family) AND (“Ambulatory Care” OR “follow cup”) AND (“Infant, Premature” OR “Premature Infant” OR “Premature Infants” OR “Preterm Infant” OR “Preterm Infants”))
Web of Science	(“Health Literacy” OR “Health Education”) AND (Family) AND (“Ambulatory Care” OR “Follow up”) AND (“Infant, Premature” OR “Premature Infant” OR “Premature Infants” OR “Preterm Infant” OR “Preterm Infants”)
LILACS/BVS	*(TW: “Letramento em Saúde” OR “Health Literacy” OR “Alfabetización en Salud” OR “Compétence informationnelle en santé” OR “Educação em Saúde” OR “Health Education” OR “Educación en Salud” OR “Éducation pour la santé”) AND (TW: Família OR Family OR Familia OR Famille) AND (TW: “Assistência Ambulatorial” OR “Ambulatory Care” OR “Atención Ambulatoria” OR “Soins ambulatoires” OR “Follow up”) AND (TW: Criança OR Child OR Niño OR Enfant OR Children) AND (TW: “Recém-Nascido Prematuro” OR “Infant, Premature” OR “Recien Nacido Prematuro” OR Prématuré OR “Bebê Prematuro” OR “Bebês Prematuros” OR Prematuro OR “Premature Infant” OR “Premature Infants” OR “Preterm Infant” OR “Preterm Infants”)*

* *BVS - Biblioteca Virtual em Saúde;*

** *Medical Literature Analysis and Retrievel System Online.*

Searches were performed for articles published between the years 2012 and 2022, in English, Spanish, and Portuguese languages, in the electronic databases MEDLINE via PubMed, Embase, Cochrane Library, Scopus, Web of Science, and in LILACS via the Virtual Health Library (BVS), for both genders (female and male), in the age groups: newborn: birth to 1 month; infant: 1 to 23 months; and preschool: 2 to 5 years. The search was conducted in September 2022 and updated in September 2023. The choice of this temporal cut-off was to map knowledge in a more recent period, considering advances in health care for prematurely born children. These advances have determined a profile of graduates with specific demands, which have varied over time according to the life context, including factors related to caregivers and care decisions.

The literature identified in all databases was imported into the Rayyan software, where duplicate articles were removed. Rayyan was then used in the selection process. Initially, two independent researchers reviewed the titles and abstracts for inclusion. These two reviewers conducted an initial selection of 10% of the publications, applying the inclusion and exclusion criteria. Subsequently, a review of conflicting decisions was conducted to develop an understanding of the inclusion and exclusion criteria and improve agreement. After reviewing 50% of the cases, new selection checks were made to resolve conflicts and proceed with the selection of the remaining publications. At this stage, an external researcher independently resolved conflicts that had not been consensual between the two initial reviewers.

After the selection based on abstracts, the same initial reviewers checked the full texts of the publications included in the initial phase. Again, two independent reviewers analyzed 10% of the articles for inclusion, met to resolve conflicts, and refined the application of inclusion and exclusion criteria. In cases of disagreement, a third researcher resolved conflicts. The same selection process was repeated for the first 50% of the records and again for the rest of the articles. The publications identified from the full reading became part of the final sample. A reverse literature search was also conducted based on the references presented by the articles included in this review.

### Data analysis

The selection of eligible articles was conducted by two independent reviewers who extracted the data, read, and analyzed the full texts. In case of divergence between the two primary reviewers, a third reviewer was consulted to decide on the inclusion of data. A preliminary data extraction tool was created based on the article’s objectives, the PCC, and the research question. The articles and their bibliographic information were exported to the MaxQDA software. With the assistance of this tool, three researchers conducted an initial review of the articles. Initially, there was a refinement of the data extraction tool to better meet the study’s objectives (as shown in Chart 1). A final sample of articles was divided between two researchers, who applied the data collection instrument. Data extraction conflicts were resolved by the researchers, and the extraction method was refined. Similarly, to the previous stage, a third researcher resolved conflicts that occurred during extraction.

**Figure 1:**
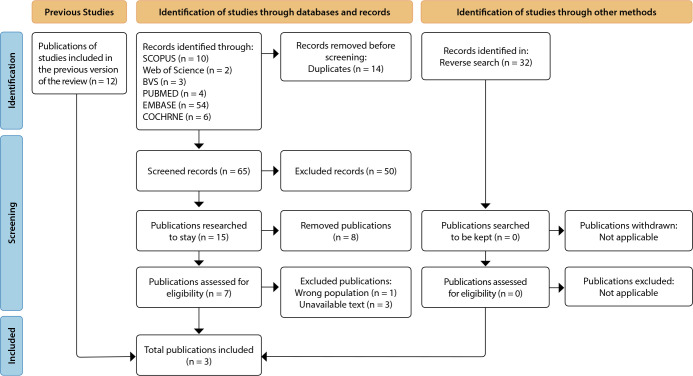
Flowchart for new systematic reviews incorporating searches in databases, protocols, and other sources, according to PRISMA-ScR, Belo Horizonte, Minas Gerais, Brazil, 2023

The search strategy results were presented according to the PRISMA-ScR (Figure 1), with the development of a diagram and its corresponding description. The results obtained from the articles were presented to address the objective and research question.

## RESULTS

For the development of the review protocol, initially, twelve theoretical frameworks were considered. However, none of them persisted for the continuity of the research. Subsequently, with the identification of studies through databases, we found: 10 studies in the SCOPUS database; 2 in the Web of Science database; 3 in the BVS database; 4 in the PubMed database; 54 in the EMBASE database; 6 in the COCHRANE database. With a total of 79 studies, a duplicate check was conducted with the assistance of the Rayyan software. Fourteen duplicate studies were identified and removed, leaving 65 articles for analysis.

In the initial selection, based on the reading of titles and abstracts, 50 records deemed inappropriate for the inclusion criteria were excluded. Thus, in the end, we had a total of 15 studies. A new analysis was performed to select eligible publications, and eight records were excluded. Among the seven possible records, one was excluded because the population did not align with that defined for this review, one presented a different context than defined, and two were excluded for not having the full text available. Thus, we concluded with three publications included, as observed in the flowchart represented in Figure 1.

A reverse search was conducted from the three selected publications, checking the references of each for the identification of potential literature. No records were included in this process.

The search was updated in September 2023, and during this process, a new article was found that did not meet the inclusion criteria for the study.

### Sources of data found

The three articles included in this review are presented in Chart 3, with their identification code, year and country of publication, study type, and participant characteristics. Concerning the context, the studies were conducted in units for the follow-up of premature neonates. Regarding the concept, one^(22)^ addressed the use of the ezParent program along with links to instruct caregivers, another^(23)^ used a caregiver education protocol to implement the Kangaroo Mother Care Method through videos and pamphlets, and the third^(24)^ implemented the use of learning portfolios. The synthesis of the results is presented in Chart 4.

**Chart 3:** Characterization of selected studies, Belo Horizonte, Minas Gerais, Brazil, 2023

Code	Year	Country	Design	Participant Characteristics
A1^(22)^	2020	United States of America	Descriptive study of a single-cohort design in a mixed methods approach.	10 countries with infants weighing less than 1500g at birth (<1500g) and who were 20-24 months corrected age.
A2^(23)^	2021	India	Prospective cohort study.	Caregivers of preterm newborns; 88 in the control group and 92 in the study group.
A3^(24)^	2018	China	Quasi-experimental design	52 mothers with preterm infants; 26 in the control group and 26 in the experimental group.

**Chart 4:** Results of selected studies, Belo Horizonte, Minas Gerais, Brazil, 2023

Code	Approach to Health Literacy	Resources Used	Desfechos
A1^(22)^	Investigation of parents’ learning regarding care strategies and practical application in their lives after intervention through telephone contact.	ezParent Program: Teaching caregivers’ behavioral strategies to be used with the child. It includes videos, knowledge questions, reflections on the topic, session activities, and practical exercises. Telephone contact is made to monitor the needs and skills acquired.	Potential for the Use of the Program with Phone Calls to be Implemented in the Clinical Setting. Teaching Program via Phone Calls has the Potential for Incorporation into Clinical Practice: A teaching program conducted through phone calls has the potential to be incorporated into clinical practice.
A2^(23)^	Evaluation of how the education of mothers and family members about the Kangaroo Mother Care Method (KMC) influenced its usage in the Neonatal Intensive Care Unit (NICU) and at home.	Kangaroo Mother Care (MC) Education Protocol: Implemented during outpatient follow-up after discharge. It involves individual counseling, videos, distribution of brochures about the benefits and procedures of MC, and group discussions.	Implementation of the Protocol Resulted in: Early initiation and increased duration of Kangaroo Mother Care (MC); Increased exclusive breastfeeding rates and involvement of other family members.
A3^(24)^	Method to enhance the knowledge and skills (health literacy) necessary for caring for preterm infants, as well as promoting caregivers’ self-confidence.	Preterm Baby Care Learning Portfolio (PICLP): Includes a list of learning tasks and self-assessment methods. Follow-up sessions are conducted after each learning task.	Knowledge and skills in caring for preterm babies improved for mothers: Both knowledge and skills, along with confidence, improved in both groups after the intervention. The experimental group showed greater improvement than the control group by the post-test.

### Review of findings

The results of the studies are presented in Chart 4, indicating the authors’ approach to the health literacy concept, the resources used to promote health literacy in caregivers of prematurely born children, and the outcomes achieved.

## DISCUSSION

The analysis of the reviewed studies enabled the mapping of available evidence on the resources used to promote health literacy among caregivers of prematurely born children in outpatient follow-up. This involved identifying the use of digital resources such as mobile applications, phone calls, and videos, as well as strategies like individual counseling, group discussions, and the use of educational pamphlets.

In the planning of the search strategy and construction of PRISMA, it was observed that there is a scarcity of publications on the proposed topic for this review. The studies had their publications concentrated in the last five years, indicating that the theme has been recently investigated. Additionally, the research was conducted by the three most populous countries in the world^(24)^, located in North America^(22)^ and Asia^(23-24)^. This suggests the need for an approach in other countries with diverse social and cultural contexts, such as Brazil, which lacks studies focused on promoting health literacy among caregivers of prematurely born children attending outpatient clinics.

In none of the three included publications did the authors define the concept of “health literacy”. Instead, they practically applied it through health education actions. According to the theoretical framework, health literacy “implies obtaining a level of knowledge, personal skills, and confidence to act to improve personal and community health, changing personal lifestyles and living conditions”^(1)^. The WHO emphasizes that by improving people’s access to health information and their ability to use it effectively, literacy empowers and expands personal, social, and cultural development^(1)^. Since none of the authors established the concept, it was necessary to go beyond the definition and investigate how the authors addressed literacy in the studies.

The presented studies utilized health education as a resource for promoting health literacy, including the ezParent program^(22)^, the Kangaroo Mother Care education protocol^(23)^, and learning portfolios^(24)^. These educational strategies contribute to health literacy by providing information and skills related to the care of prematurely born newborns.

The learning portfolio presented a differential in its planning, as study participants received a semi-structured portfolio in which they could plan their study as needed. This method enhances health literacy, as caregivers become co-responsible for developing the resource, making it more accessible for learning.

Health education can be recognized as a resource that promotes family literacy, aligning with other actions and contributing to the set of skills necessary for decision-making in health^(25)^. Thus, it can be inferred that the results of the studies addressed strategies aimed at promoting literacy, even though the concept was not explicitly stated. Resources providing necessary information to impact health decisions were demonstrated in the cited articles, highlighting the presence of literacy.

Pediatric educational programs associated with technology have been used as strategies for health literacy, demonstrating the feasibility of using the internet as a means of health promotion and information access^(26)^. Beyond the outpatient context, a study conducted with parents and nurses in the Neonatal Intensive Care Unit (NICU) aimed to explore their opinions on the development of a digital educational program to meet educational needs. The need for easy access to a mobile device or early availability was revealed, along with addressing topics that are part of the family’s everyday life, such as information about equipment and places to turn in case of complications^(27)^.

In the literature, there are studies addressing the promotion of literacy, especially in individuals with Diabetes Mellitus^(28)^, pregnant women^(29)^, and oral health^(30)^. It was found that the use of resources such as expository dialogue, question and answer games, mobile applications^(27)^, group discussions, and remote meetings^(29)^ led to an expansion of knowledge, directly influencing literacy. The use of pamphlets^(30)^ did not yield satisfactory results, as the language was difficult to comprehend, requiring adaptation for the population to have access.

It is known that health literacy is part of the social determinants that impact the health gains of the population^(31)^. However, there is a lack of studies addressing health literacy in the context of the health of premature children. Considering the unfavorable outcomes found in the context of this population, addressing the promotion of literacy in caregivers of these children is necessary.

### Study limitations

This review has limitations, with the main one being the heterogeneity of the profiles of caregivers and children included in the evaluated studies. This variability in participant characteristics should be considered when analyzing the results of this review, as the development of the set of skills that forms the basis for health literacy can be influenced by factors such as caregivers’ socio-economic conditions (education, age, support network), as well as the specific demands of caring for children with different profiles, risks, and health conditions.

### Contributions to Nursing and Health

Our results emphasize the importance of incorporating discharge planning from the moment of patient admission, highlighting the crucial role of nurses in this process. This approach contributes to a qualified discharge, ensuring the continuity of care at home, which, in turn, enhances better health outcomes and reduces care-related expenses. Thus, the nursing team plays a crucial role in promoting health education and building resources that can be applied to families during the transition from the Neonatal Intensive Care Unit (NICU) to home, as well as in post-discharge follow-up.

Furthermore, the findings of this study highlight the need for future research to investigate the impact of resources used in promoting the literacy of caregivers of prematurely born children in the outpatient context. This can provide valuable insights for improving nursing practice and contribute to more effective care for this vulnerable population.

## CONCLUSIONS

Technological devices and guidance from healthcare professionals were the primary resources used by families to promote their health literacy. One of them, the ezParent program, showed potential for application in clinical practice, although its use depends on access to technological resources, such as tablets, for implementation. On the other hand, the Kangaroo Mother Care (MC) protocol proved effective in promoting literacy when combined with other strategies, such as individual counseling and follow-up sessions. Additionally, learning portfolios proved effective for literacy, especially due to patient involvement in the construction process, promoting autonomy.

This highlights the importance of investing in strategies that promote the literacy of caregivers of prematurely born children in the outpatient context, as this can result in benefits such as early diagnosis, systematic monitoring, and early intervention. It is essential to note that the information presented is subject to modifications as advances in scientific discoveries occur.
